# The effect of telephone counseling based on Orem’s model on adherence to treatment and resilience of patients with coronary angioplasty: a randomized clinical trial

**DOI:** 10.1186/s12872-023-03529-9

**Published:** 2023-10-04

**Authors:** Khatereh Rostami, Mahsa Maryami, Masoume Rambod

**Affiliations:** 1 Community Based Psychiatric Care Research Center, Nursing and Midwifery School, Shiraz University of Medical Sciences, Shiraz, Iran; 2 Student Research Committee of Shiraz University of Medical Sciences, Shiraz, Iran

**Keywords:** Orem Self Care Model, Treatment adherence, Resilience, Coronary angioplasty, Tele-nursing

## Abstract

**Background:**

This study aimed to determine the effect of telephone counseling based on Orem’s Self-Care Model on adherence to treatment and resilience of patients with coronary angioplasty.

**Methods:**

This randomized clinical trial was performed on 80 patients in the Cardiac Intensive Care Unit of Shiraz University of Medical Sciences. Patients were randomly divided into two groups of 40 (intervention and control). Questionnaires on adherence to treatment of chronic patients and resilience for patients with cardiovascular and respiratory diseases were filled out before and 8 weeks after the intervention. In the intervention group, the telephone call schedule consisted of three calls per week for 8 weeks.

**Results:**

Before the intervention, no significant difference was found between the groups about adherence to treatment and resilience. However, after the intervention, a significant difference was found between the groups as to adherence to treatment and resilience (P < 0.001).

**Conclusion:**

Nursing consultation using telephone calls based on Orem’s model increases the adherence to treatment and resilience of patients undergoing coronary angioplasty. Telephone counseling can help the patients adhere to their treatment plans and develop resilience skills.

## Introduction

Cardiovascular diseases are predicted to be the main cause of death and disability in the world by 2030 [1]. It shows that recent surgical and pharmacological interventions have reduced mortality from heart diseases [2]. One of the treatment options available for coronary artery disease is percutaneous coronary intervention (PCI) [3]. The use of PCI has increased steadily over the past decade. When the treatment is successful, the patient may even be discharged on the same day as the procedure. Shorter hospitalizations are cost-effective, but the responsibility for care transfers quickly to the patients, who may mistakenly believe that they have fully recovered. This can lead to reduced understanding of the risk factors and the seriousness of coronary artery disease [4].

One of the behaviors related to the disease and self-care is patients’ adherence to a treatment regimen that can predict successful treatment and reduce the severity of the disease and its complications [5]. Adherence to treatment consists of adherence to medication and a healthy lifestyle, including a healthy diet, physical activity, lack of smoking, and lack of alcohol consumption [6]. Adherence to treatment reduces cardiovascular mortality and re-hospitalization [7]. However, failure to follow treatment has been reported in 60% of patients with cardiovascular disease [8]. Adherence to treatment in coronary angioplasty may be a seriously stressful event for patients [9]. Resilience may be helpful in promoting mental health and coping with the patient’s challenges [10].

Resilience is a good adaptive indicator that is used when faced with catastrophic disasters such as traumatic events [11]. Resilience means the ability of individuals to maintain healthy mental development in the face of adverse factors [9]. The three main characteristics of resilience include the characteristics of returning to previous functional levels, increasing the level of adaptation, and maintaining mental health. Resilience in people with chronic physical illness results in self-care, adherence to treatment, increase in empowerment and self-efficacy, reduction of anxiety, and increased optimism [12].

Resilience refers to the ability to bounce back from times and regain strength by adapting and maintaining an attitude even when facing difficult situations in life [13]. Self-adherence entails engaging in practices that contribute to one’s health and wellbeing [14]. Dorothea Orem’s nursing theory explores the ways individuals care for themselves, emphasizing their capacity to engage in activities that promote emotional wellbeing. In Orem’s theory, self-adherence and resilience are connected as they both revolve around an individual’s ability to effectively nurture him/her and maintain health [15].

The high prevalence and consequences of coronary artery disease, as a chronic, progressive, and disabling disorder that reduces physical ability, impair the individual and social relationships, and economic problems of the patients, all necessitating the need for nursing interventions [16]. These interventions are necessary for modifying health behaviors, including adherence to treatment and a healthy lifestyle, so that the individual can achieve self-care. Self-care has been defined as the process of taking care of one with behaviors that promote health and active management of illness when it occurs [17]. Self-care is one of the main elements and cornerstones of the treatment of cardiovascular patients. Thus, self-care activities may have a positive effect on adherence to treatment and play an important role in reducing recurrent hospitalizations of cardiovascular patients [18].

One of the most complete clinical guidelines for planning and implementing self-care principles is the Orem’s self-care model. Orem’s theory is about how individuals look after themselves; accordingly, taking care of yourself means doing things by yourself to keep your body and mind healthy and happy [15]. In this model, Orem considers the care receiver to be a person who is unable to take care of himself/herself independently due to health limitations. Orem has designed three types of care systems based on the condition and needs of patients when they deviate from their health: complete compensation system, partial compensation system, and supporting education system [19]. When the patient is able to take care of self but needs the nurse’s assistance in making decisions, controlling behavior, and acquiring knowledge and skills, the supporting education system is used. In this system, the nurse has more of an educational and advisory role [20]. Self-care slowly helps the patients to have more control over their daily lives and cope with their social functioning, while inadequate self-care leads to poor health outcomes and frequent hospitalizations [21].

Remote nursing counseling using a mobile phone not only is cost-effective and provides the uniqueness of each patient’s care, but also facilitates and improves access to effective health care [22]. A systematic review and meta-analysis indicated that smartphone apps, telemonitoring, and clinician-driven SMS as a novel method had a valuable adjunct in cardiovascular diseases management. They reported that mobile phone intervention reduced the rate of hospitalization in heart failure patients. Moreover, they indicated that this intervention decreased systolic blood pressure in hypertension patients [23]. Another study showed that mobile apps might improve medication adherence in hypertension patients [24]. In a review study by Adler et al., it was shown that mobile text messaging had a positive effect on secondary prevention following cardiovascular disease treatment, but there is insufficient evidence to confirm its effectiveness on adherence to medication [25]. Using mobile lifestyle counseling as a feasible intervention may be effective in cardiovascular diseases [26]. Lifestyle counseling improved the health behavior and decreased the risk of cardiovascular disease [27, 28].

Upon reviewing the literature, it was found that education played a crucial role in enhancing individuals’ resilience and equipping them with necessary skills to effectively confront the challenges of life in a positive manner [10]. In previous studies, telephone consultation has not been done based on the patients’ needs assessment, and the number of calls and duration of telephone consultation have been limited [29]. In this study, based on the assessment of the patients’ needs, we increased the frequency of calls and extended the duration of communication with them. Therefore, performing interventions using telephone counseling by nurses based on the Orem self-care model may be useful in following the treatment regimen by the patients and promoting resilience in them.

This study aimed to determine the effect of nursing counseling through telephone calls based on Orem’s self-care model on adherence to treatment and resilience in cardiac patients treated with coronary angioplasty.

## Methods

### Design

The present study was a randomized clinical trial. This was registered in the Iranian Registry of Clinical Trail (Number IRCT20210303050562N1, approved 18/06/2021). (https://www.irct.ir/trial/55950). The study was conducted from July 2021 to August 2021.

### Setting

The study was performed at eight Cardiac Intensive Care Unit (CCU) wards in the hospitals affiliated with Shiraz University of Medical Sciences in 2021.

### Eligibility criteria for participants

The inclusion criteria of the study were being hospitalized post-PCI patients; ranging in age from 18 to 65 years; being able to communicate and no hearing or speech problems; having access to mobile or landline phone and ability to use it; being able to read and write; and being able to perform daily activities. On the other hand, known severe mental illness; incomplete completion of questionnaires; failure to cooperate completely; and participation in a similar intervention in the last 3 months were the exclusion criteria of the study.

**Fig. 1:**
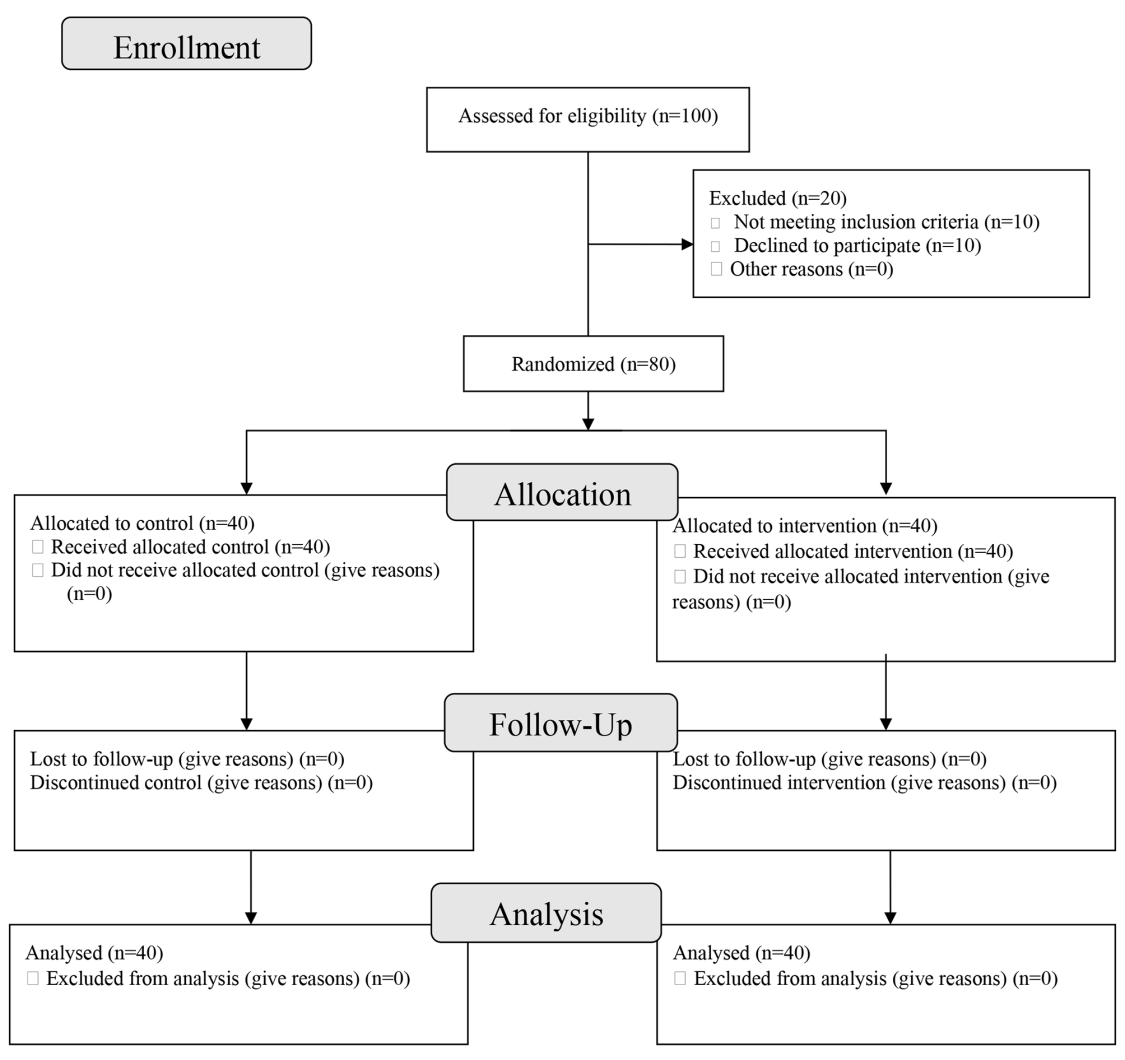
Flowchart of the study participants

As Fig. 1 shows, first, the researcher selected 100 patients from each department among eight CCU wards. Twenty subjects did not meet the inclusion criteria (n = 10) or were not willing to participate (n = 10). Then, 80 patients were enrolled in this study. After introducing himself, stating the objectives of the research, and announcing the voluntary participation of patients in the research, the researcher obtained informed written consent from the participants. They were then allocated into the intervention or the control group by block randomization. All of subjects continued the study and finished it.

It should be noted that zero drop rate and 100% follow-up in this study may be associated to using the following strategies: (1) Regular follow-up calls caused the participants to ensure their continued participation and addressed any concerns or issues they might have had, (2) Flexible titration schedule for the study medications was implemented to accommodate the participants’ needs and minimize dropout. Moreover, we understand the importance of minimizing dropout in maintaining the integrity and validity of the study. Dropout can introduce bias and affect the generalizability of the results. Regarding the selection process, we would like to clarify that the subjects were chosen based on specific criteria relevant to the study, rather than solely on the likelihood of cooperation.

### Sample size

The research sample size was determined using medcalc software and the study conducted by Akhu-Zaheya et al. (2017) [22]. Based on this study and medication adherence, µ1-µ2 = 1.05, SD = 1.3, α = 0.05, 1-β = 0.9, and drop rate = 18%, the sample size was determined 80 patients (40 in each group).

### Randomization

To conduct the randomization, we firstly selected eight CCU wards as eight separate strata. Then, using a statistical software and eight separate strata, we prepared a list of random allocation for 80 subjects. A statistician who was not a member of the research team generated the list and made sequentially numbered containers. Then, based on the random sequence that was prepared, the researcher assistant opened the containers sequentially, and the eighty patients under PCI were randomly divided into the intervention and control groups. Allocation concealment that prevents selection bias was implemented through a randomization for allocation to the two groups; we kept the random allocation sequence in a locked numbered containers, so the statistician and outcomes assessors were blind.

### Blinding

In this study, the individual who collected the data and the statistician were blind to the groups. Also, the outcomes were assessed by a blinded examiner before and 8-weeks after the intervention. Moreover, the researcher who selected the participants and allocated them to the groups was blind to them.

#### The intervention

In the interventional group, in addition to educational pamphlets and routine hospital care, telephone counseling was performed for 8 weeks. It was conducted by a MSc nurse that had 3 years of experience working as a nurse in CCU wards. Each patient gave us his/her contact number as well as that of a family member; the researcher gave them her contact number. They were provided with a schedule of telephone calls, which included 3 calls per week for 8-weeks. The average call time was 30 min. The range of contact time was from 8 A.M. to 9 P.M. In the case of any problems or questions, they were in contact with the researcher at the same time interval. The patient was referred to a physician to follow up on his/her questions and problems, if necessary.

In the first telephone call, the Orem Needs Assessment was utilized to evaluate the client’s needs during the initial telephone call in the intervention group. This program incorporates distinctive aspects, including the identification of universal and health deviation self-care requisites, as well as promotion of the individuals’ independence and active engagement in self-care for maintaining the structural and functional integrity of a person [19]. The intervention was based on the nursing process of Orem’s self-care model; based on the assessment of self-care needs (including universal, developmental, and bloom aberration needs) and self-care agency, the patients were evaluated from three aspects in the field of nursing diagnosis: nursing analysis or self-care deficit; ambition setting; and nursing arrangement architecture (including wholly compensatory, partially compensatory, and supportive–educative nursing systems) and methods of allowance (including acting, guiding, teaching, supporting, and accouterment an environment); planning; implementation; and follow-up and evaluation [30].

The care plan was designed and developed based on the goals in the form of a supporting education system that included: (1) Meeting the patient’s needs in self-care, (2) Making the patient responsible for self-care, and (3) Increasing the patient’s independence in care and his/her adaptation to the changes that have been made, etc. Therefore, the nurse here has an advisory role based on Orem’s supporting education system. For example, for a heart disease patient who has high blood cholesterol, the nurse helps the patient by educating him/her about a healthy diet such as reducing the consumption of saturated fats, losing weight, etc. by increasing awareness about their diet. Moreover, a patient with heart disease who had undergone angioplasty and his self-confidence had decreased due to the limitations, the nurse helped him to trust his abilities against the disease by focusing on the patient’s capabilities, strengthening positive behaviors, and improving the behavioral and mental performance of the patient to help him adapt to the disease and feel good despite the limitations [12]. The planned nursing measures were implemented in the form of supportive counseling; finally, evaluation was performed based on the goals of reducing needs and increasing abilities through telephone calls and repetition, and practice.

The content of telephone conversations generally included the researcher’s self-introduction, inquiring about the patient’s general health condition, giving healthcare advice, informing the patient about nutrition and medication, assessing the patient’s level of adherence to dietary and medication regimen, evaluating the behavioral objectives agreed upon by the patients and the researcher, reinforcing health behaviors, terminating the call with the words of encouragement, and arranging the next contact. During the telephone conversation, the researcher emphasized compliance with the plans and proposed possible solutions to the patient’s problems.

### The control group

The control group received routine care without any interventions. The patients received the routine care as follows: educational pamphlets and standard hospital care including education about diet, activity, medications, and educational pamphlets about disease and medicine, and the way to see a doctor at the time of discharge were presented, but no pre-planned program was performed.

### Outcomes

Data collection tools included demographic and clinical characteristics forms, adherence to treatment of chronic patients (Modanloo 2013) [31], and resilience for patients with cardiovascular and respiratory diseases (Ebadi 2016) [12]. The participants in both groups were examined for adherence to treatment and resilience at baseline and 8 weeks after the intervention.

The demographic and clinical characteristics form which was collected at a baseline included information on the patient’s age, gender, marital status, educational level, job, body mass index (BMI), hypertension, diabetes, and hyperlipidemia, family history of heart disease, and history of smoking.

### Adherence to treatment

Modanloo’s Chronic Patients Adherence Questionnaire was used to assess adherence to the treatment of heart patients in various dimensions (acceptance of medication, diet, weight control, physical activity, follow-up time for treatment, and lifestyle changes). This scale has 40 items in the form of 7 subscales in the areas of concern in treatment (9 questions), willingness to participate in treatment (7 questions), ability to adapt (7 questions), integration of treatment with life (5 questions), adherence to treatment (4 questions), commitment to treatment (5 questions), and management of treatment (3 questions). This questionnaire is scored based on 5-point Likert scale (strongly agree = 1, disagree = 2, neutral = 3, agree = 4, strongly agree = 5). The higher the total score or the score of each subscale, the higher the adherence of the respondent. In Modanloo’s study, the correlation coefficient test-retest was reported 0.87. The internal consistency was Cronbach’s alpha = 0.92 [31]. The psychometric properties of the questionnaire were determined using face, content, and construct validity as well as internal consistency, reliability, and stability (test-retest). The average content validity index of the questionnaire was 0.91 [31]. In the present study, the reliability of the questionnaire was obtained at 0.92, using Cronbach’s alpha.

### Resilience

Resilience for patients with cardiovascular and respiratory diseases was designed by Ebadi et al. in 2016. This scale has 29 items with 5 subscales in the dimensions of positive adjustment (10 questions), self-management dimension (7 questions), rational empowerment dimension (7 questions), treatment adherence dimension (3 questions), and spirituality dimension (2 questions). This questionnaire is also scored based on a 5-point Likert scale (strongly disagree = 1, disagree = 2, neutral = 3, agree = 4, strongly agree = 5). The scores range from 29 to 145, so the higher the score, the more positive the patient’s resilience. Cronbach alpha coefficient for the whole questionnaire was 0.96. Also, the intra-class correlation coefficient was 0.95, based on the result of the re-test. The psychometrics of the questionnaire was assessed using face and content validity, internal consistency, and stability. The findings showed that the resilience questionnaire for patients with cardiovascular and respiratory diseases (Ebadi 2016) was valid and had a high level of reliability [12]. In this study, the reliability of the questionnaire was estimated at 0.95, using Cronbach’s alpha.

### Ethical considerations

We obtained the code of ethics (1400.167. IR.SUMS.REC) from the Research Ethics Committee of Shiraz University of Medical Sciences; we also obtained permission from the University of Medical Sciences and related hospitals. The written informed consent was signed by the participants in the study. They were assured of the anonymity and confidentiality of information in all stages of the research and had the right to withdraw from the study at any time.

### Statistical analyses

To describe the findings, we used the mean, frequency percentage, variance, and standard deviation. To determine the confounders and other factors that might be associated to adherence to treatment and resilience, we used multiple linear regression analysis. It showed that age, gender, marital status, educational level, job, BMI, having hypertension, diabetes, and hyperlipidemia, family history of heart disease, and history of smoking were not associated to adherence to treatment and resilience. The Kolmogorov-Smirnov test was used to check the normality of data distribution. The studied samples did not have a normal distribution. Statistical analysis (Chi-square test), as well as non-parametric tests (Wilcoxon, Mann-Whitney test), were used for intergroup and intragroup comparisons. The significance level in statistical tests was considered 0.05, and SPSS statistical software version 23 was used to analyze the data.

## Results

### Sample characteristics

The final sample of 80 respondents was randomly assigned into two groups of 40, intervention and control subjects. The youngest participant in this study was 41 years old and the oldest was 65 years old, and the mean age of the participants was 56.60 ± 5.546 years. According to the Mann-Whitney test, there was no significant difference between the two groups in terms of age (P = 0.68). The majority of the subjects in both groups were male (61.3%) and married (83.8%). Most participants in the intervention and control groups (52.5%) had a diploma and were retired (32.5%). The results of the chi-square test showed that there was no significant difference between the two groups in terms of demographic characteristics including gender, marital status, education level, and job (P > 0.05). Details of the respondents are presented in Table 1.

The BMI of the participants was reported 25.65 ± 2.896. The means of their BMI were 25.85 ± 2.88, and 25.43 ± 2.92 in the intervention and control groups, respectively. The results showed no significant difference between the two groups in terms of BMI (Z=-4.63, P = 0.67) (Table 2).

According to the results shown in Table 3, most of the participants (61.3%) were hypertensive. The majority of them (75%) were nondiabetics. Most of the subjects in both study groups had a history of hyperlipidemia (53.8%). In addition, most of the patients had a family history of heart disease (53.5%) and a history of smoking (46.3%). The results showed no significant difference between the two groups in terms of clinical characteristics (P > 0.05).

As shown in Table 4, the results showed that there was no statistically significant difference between the mean score of adherence to treatment in the intervention and control groups before the intervention (P = 0.06). However, the results showed that the mean score of adherences to treatment by the patients was significantly different in the two groups after the intervention (P < 0.001). There was a significant difference in the mean score of adherence to the treatment of patients in the intervention group before and after the intervention (P < 0.001). However, there was no statistically significant difference between the mean score of adherence to treatment in patients in the control group before and after the intervention (P = 0.31).

This study indicated that there was no statistically significant difference between the resilience mean scores of the two groups before the intervention (P = 0.92). On the other hand, the resilience mean score of patients in the intervention and control groups was statistically significant after the intervention (P < 0.001). The mean score of resilience inpatients of the intervention group before and after the intervention was significantly different (P < 0.001). There was a statistically significant difference in the resilience mean scores of patients in the control group before and after the intervention (P = 0.02); however, this difference was reduced in the control group.

**Table 1:** Determination and comparison of demographic characteristics in the intervention and control groups

Demographic profile	Classification	Group		Test† P- value
		Control n (%)	Intervention n (%)	
**Age**	41–50	8(20)	6(15)	χ^2^=-0.40 P = 0.68
	51–65	32(80)	34(85)	
**Gender**	Man	23(57.5)	26(65)	χ^2^ = 0.47 P = 0.49
	Female	17(42.5)	14(35)	
**marital status**	Married	33(82.5)	34(85)	χ^2^ = 0.09 P = 0.76
	Single	7(17.5)	6(15)	
**Education rate**	High school	13(32.5)	11(27.5)	χ^2^ = 0.45 P = 0.79
	Diploma	21(52.5)	21(52.5)	
	Bachelor’s degree and higher	6(15)	8(20)	
**Job**	Retired	9(22.5)	17(42.5)	χ^2^ = 6.28 P = 0.17
	Employee	7(17.5)	8(20)	
	Free	4(10)	5(12.5)	
	Unemployed	5(12.5)	1(2.5)	
	housewife	14(35)	9(22.5)	

†Chi-square (χ^2^)

**Table 2:** Determination and comparison of demographic in the intervention and control groups

Demographic profile	Total Participants M(SD)	Intervention group M(SD)	Control group M(SD)	P- value
**Body Mass Index (BMI)**	25.65(2.89)	25.85(2.88)	25.43(2.92)	Z=-4.63* P = 0.67

* Mann-Whitney U Test (Z)

**Table 3:** Determination and comparison of clinical characteristics in the intervention and control groups

Clinical characteristics	Classification		Total Participants n (%)	group		Test† P- value
				Control n (%)	Intervention n (%)	
**blood pressure**	Yes		49(61.3)	23(57.5)	26(65)	χ^2^ = 0.47 P = 0.49
	No		31(38.8)	17(42.5)	14(35)	
**Diabetes**	Yes		20(0.25)	11(27.5)	9(22.5)	χ^2^ = 0.26 P = 0.6
	No		60(0.75)	29(72.5)	31(77.5)	
**Hyperlipidemia**	Yes		43(53.8)	21(52.5)	22(55)	χ^2^ = 0.05 P = 0.82
	No		37(46.3)	19(47.5)	18(45)	
**Family history of heart disease**	Yes		42(53.5)	20(50)	22(55)	χ^2^ = 0.2 P = 0.65
	No		38(47.5)	20(50)	18(45)	
**Smoking**	Yes	Cigarettes	21(26.3)	9(22.5)	12(30)	χ^2^ = 0.58 P = 0.44
		Hookah	13(16.3)	7(17.5)	6(15)	χ^2^ = 0.09 P = 0.76
		Alcohol	1(1.3)	1(2.5)	0(0)	χ^2^ = 1.01 P = 0.31
		Other drugs	2(2.5)	1(2.5)	1(2.5)	P = 1
		Total	37(46.3)	18(45)	19(47.5)	χ^2^ = 0.05 P = 0.82
	No		43(53.8)	22(55)	21(52.5)	

†Chi-square (χ^2^)

**Table 4:** Comparison of the mean score of adherence to treatment and resilience of the intervention group compared to the control group before and after the intervention

		Intervention group	Control group	Test P -Value
		M(SD)	M(SD)	
**Adherence** **to treatment**	Before	22.47(4.53)	20.70 (5.01)	Z †=-1.86 P = 0.06
	After	38.64(3.76)	21.13(4.15)	Z†= -7.70 P < 0.001*
	Wilcoxon	Z= -5.51 P < 0. 001*	Z =-1 P = 0.31	
**Resilience**	Before	31.33(6)	30.99(6.50)	Z†= 0.09 P = 0.92
	After	43.51(8.62)	27.78(5.19)	Z†= -6.78 P < 0.001*
	Wilcoxon	Z= -5.21 P < 0.001*	Z= -4.55 P = 0.02	

* Indicates significance level of p < 0.05

† Mann-Whitney U Test (Z)

## Discussion

The results of the study showed that there was a difference between the two groups in terms of adherence to treatment and resilience after the intervention. The results of the present study also showed that the mean scores of adherence to treatment and resilience were significantly different in the intervention group before and after the intervention, but they were not significantly different in the control group. According to the findings, adherence to treatment by patients after the intervention was more than that in pre-intervention treatment. Consistent with the present study, Oscalices et al. reported the effect of telephone follow-up and discharge instructions on the adherence to treatment of patients with heart failure [32]. Based on the findings of a review study conducted by Younas et al., an educational program based on the Orem’s model and the preparation of educational materials based on the needs and beliefs of the patients in a comprehensible way could improve the patients’ adherence to the treatment [33]. In a similar study by Dale et al., text messaging combined with website support for improving adherence to treatment in heart disease patients showed that text messaging increased adherence to treatment by patients for over 3 months, but it had no effect over 6 months [34]. The probable reason for this discrepancy may be the patients’ frustration with treatment following the chronicity of the disease. In addition, the follow-up of the proposed treatments was not based on the patients’ needs assessment. According to the results of the study carried out by Furuya et al., telephone counseling based on the Orem’s model is one of the strategies that can be used to improve self-care in chronic patients [35]. In the present study, telephone counseling was designed and implemented based on the patients’ needs assessment and Orem’s care plan program in the form of an educational-supportive nursing system, which has led to increased self-care behaviors and improved patient adherence to treatment. Based on the educational support system of the Orem’s model, with appropriate guidance and support for self-care behaviors and through education based on non-drug management strategies in heart patients, the researcher aimed to create new self-care behaviors in patients with heart disease in 8 weeks. Finally, the support of patients through telephone calls and repeated counseling helped to establish new self-care behaviors and improved adherence to treatment.

The results of this study showed a significant difference between the two groups in terms of resilience after the telephone counseling based on Orem’s model. According to the results, in the intervention group, the mean resilience after the intervention was significantly higher than that before the intervention. However, in the control group, the mean score of resilience decreased after the intervention compared to before it. Consistent with the present study, Studies have shown that cardiovascular diseases have lower scores in resilience dimensions than healthy people. Also, resilience decreases during time [36].According to the results of a study carried out by Ghezelsefloo et al. indicated the effect of educational interventions on increasing the level of resilience in the elderly patients. In this study, the intervention group experienced less stress after training, compared to the control group [37]. According to a study, the level of anxiety and depression in cardiac patients increases after coronary angioplasty, which leads to an increase in the burden of negative and psychological emotions in patients [38]. Therefore, we expected the patients’ resilience to decrease overtime, as it occurred in our control group. In fact, resilience helps the patients recover by creating a positive attitude in them and influencing their mood. Therefore, using some interventions helps the patients to overcome problems by increasing resilience skills and self-confidence and increase the ability to effectively implement their care [38]. Accordingly, nurses can improve the patients’ resilience by cultivating and improving self-efficacy and changing the patients’ attitudes about the discomfort of coronary artery angioplasty through self-care programs and increasing their trust in treatment in the face of adverse disease effects [39, 40]. According to the results of a study carried out by Momenabadi et al., education based on the Orem’s self-care model was effective in improving the resilience of patients with multiple sclerosis. This study showed that people with high levels of resilience exhibited more flexible behaviors in stressful situations. These flexible behaviors provide more insight into situations such as anger management, stress management, problem-solving, etc. [41]. A study has shown that the Orem’s self-care model addresses all aspects of self-care (physical, mental, social), and patients are properly educated based on this model. They accept life with their illness and learn how to cope with difficult situations, and by using active coping skills such as self-care behaviors, they reduce the feeling of illness in themselves and increase resilience [42].

In this study, the authors devised a care plan for individuals with heart conditions based on their self-care requirements. The plan included educating and guiding patients about their condition and the way to manage their medications as well as assisting them in developing and maintaining independence in self-care. The healthcare team worked closely with patients to identify any gaps or shortcomings in their self-care skills offering activities and interventions to address those areas effectively. Early encouragement of self-care was emphasized to facilitate the patients’ attainment of independence.

Furthermore, nurses played a role in educating the patients about the techniques and strategies for self-care. They collaborated with patients to create care plans that catered specifically to their needs and goals. Emotional support was also provided throughout the process. Various efforts were made to meet these care needs while promoting autonomy, such as establishing a supportive environment that encouraged active patient participation in their own care activities. Regular progress assessments were conducted, allowing for adjustments to the care plans in order to instill confidence in patients.

Using a specific questionnaire for measuring adherence to treatment in heart patients was one of the strengths of this study. However, it is suggested in another study that the indirect adherence to treatment should be assessed. For example, adherence to medications such as clopidogrel would be assessed by the number of drug administration, skipping administration, and incorrect times and doses. Adherence to diet would indirectly be assessed by some laboratory tests such as the level of cholesterol, triglycerides, LDL, and HDL. Moreover, adherence to the follow-up time for treatment would be assessed by the number of physicians missed.

## Conclusion

The results of the present study showed that nursing counseling by telephone based on the Orem’s model increased the adherence to treatment and resilience of patients who had undergone coronary angioplasty. Therefore, mobile counseling, as an inexpensive method, can change the focus of the treatment and care from the clinic to the patient’s daily life. The widespread use of mobile phones has provided a promising opportunity to improve the care and self-management of heart patients. Nurses can also deal with the complications and effects of the disease by learning resilience methods and changing the patients’ attitudes about the stress caused by coronary angioplasty, thereby improving resilience in patients.

### Practice implications

According to the results of this study and the effect of telephone counseling on adherence to treatment and resilience of patients after angioplasty, it is recommended that all patients in this ward should be considered counselors who can counsel these patients by telephone. Also, a center in the hospital should be designed as a call center where counselors can monitor the patient’s condition at home. Depending on the content of the course (explanation of decision styles, problem-solving steps, anger management relaxation techniques, stress management techniques, etc.), face-to-face meetings could be appropriate along with telephone counseling. Also, the use of videos containing this content might be possible for patients. It is better than telephone consultation, so it is suggested that these points should be considered in future studies.

## Data Availability

The data that support the findings of this study are available from [Khatereh Rostami], but restrictions apply to the availability of these data, which were used under license for the current study, so they are not publicly available. Data are, however, available from the authors upon reasonable request and with permission of [Khatereh Rostami].
